# Subjective Cognitive Decline and Excessive Spending in WALLET study participants

**DOI:** 10.1093/geroni/igaf122.2873

**Published:** 2025-12-31

**Authors:** Emily Flores, LaToya Hall, Peter Lichtenberg

**Affiliations:** Wayne State University, Detroit, Michigan, United States; Wayne State University, Detroit, Michigan, United States; Wayne State University, Detroit, Michigan, United States

## Abstract

Older adults experiencing cognitive decline are at an increased risk of financial vulnerability, yet self-awareness of this relationship remains unclear. This study examines the association between self-reported cognitive decline and excessive spending, exploring whether individuals who perceive memory loss recognize its financial impact. Data were drawn from a sample of 83 older adults in the WALLET study who shared 12 months of their checking account statements (M = 73.54, SD = 8.05), who were experiencing early memory loss. Twenty-five participants reported that memory loss was impacting their financial decision making and 58 said memory loss had no impact on their financial decision-making. Excessive spending was significantly more prevalent in those who reported that memory loss was impacting their financial decision-making, X² = 9.285, p < .01, d = .34. The group reporting that memory loss impacted financial decision-making also had significantly higher financial exploitation vulnerability scores (FEVS) than those whose memory decline was not reported to impact financial decision making, ( t = -2.609, p < .05, d = -.55). This pattern remained even excluding the cognition-related FEVS item, t = -2.087, p < .05, d = -.52. Age and education were not significantly different between groups.The results suggest that awareness of how early memory loss impacts financial decision-making might be a key factor in risk of excessive spending and financial exploitation vulnerability. Future research will examine the utility of a 3-point scale Subjective Cognitive Decline-Financial measure (i.e. no cognitive decline, cognitive decline only, cognitive decline impacting financial decision making).

